# Epidemiologic Research on Healthy Life Expectancy and Proposal for Its Extension: A Revised English Version of Japanese in the Journal of the Japan Medical Association 2019;148(9):1781-4

**DOI:** 10.31662/jmaj.2020-0027

**Published:** 2020-07-07

**Authors:** Ichiro Tsuji

**Affiliations:** 1 Division of Epidemiology, Department of Health Informatics & Public Health, School of Public Health, Tohoku University Graduate School of Medicine, Sendai, Japan

**Keywords:** Healthy life expectancy, Epidemiology of aging, Health Japan 21, Healthy longevity society

## Abstract

Healthy life expectancy is an indicator that represents a composite of data on mortality and health status and is defined as the average number of years that a person can expect to live at a certain level of health. To extend people’s healthy life expectancy, my colleague and I conducted a variety of epidemiologic research based upon community-based cohort studies and intervention trials. The findings from our prospective cohort studies included blood pressure reference values measured at home, green tea health benefit, Japanese dietary pattern, and feeling *ikigai* (a sense of life worth living) at daily life.

Based upon these evidence, I have made some proposals toward extension of healthy life expectancy. In 2011, as the Chair of the Planning Committee for the Next National Health Promotion of Ministry of Health, Labor and Welfare (MHLW), I proposed that the Health Japan 21 (second term) should aim to extend healthy life expectancy to exceed the number of years extended in the total life expectancy, thus compressing the duration to be spent in an unhealthy state (compression of morbidity). In the interim evaluation of the Health Japan 21 (second term) in 2018, we were able to demonstrate that this goal is being achieved. Compared with 2010, in 2016, the increase in healthy life expectancy (1.72 years in men and 1.17 years in women) was higher than that of total life expectancy (1.43 years in men and 0.84 years in women). As a result, the duration to be spent in an unhealthy state was reduced by 0.29 years in men and by 0.33 years in women. It is important to note that Japan is the only country that has made progress in achieving compression of morbidity at the national level. We need to maintain this momentum of compressing morbidity.

## Introduction

Rapid advances in medical care in the latter half of the twentieth century have contributed to the extension of life expectancy of the population. Consequently, life expectancy at birth of the Japanese population increased by approximately 20 years for both men and women, from 58.0 and 61.5 years in 1950 to 77.7 and 84.6 years in 2000, respectively.

What do we expect will happen in regard to the life expectancy in the future? According to the Population Projection by the Japanese government in 2017, life expectancy at birth of men and women in 2050 is expected to be 84.0 and 90.4 years, respectively ^(1)^. These estimates represent that life expectancy at birth of Japanese people would increase only by 6 years over the 50-year period from 2000 to 2050, indicating that the increase during the first half of the twenty-first century would be by far smaller than that we have seen in the latter half of the twentieth century.

Conversely, the rapid increase in life expectancy has resulted in the growing number of older individuals with dementia and those requiring long-term care, as well as anxiety and a sense of isolation among the aged population. We may call this situation as the cost of longevity.

This phenomenon calls for a paradigm shift in medicine to focus on “adding life to years (enhancing quality of life in the old ages)” instead of “adding years to life (prolonging longevity).” Conventionally, life expectancy was considered the most important indicator of health status. However, this only asks how many years a person is expected to live, regardless of whether he or she is healthy or ill. In modern society, the most important question to ask is how many years a person is expected to live a healthy and independent life. Healthy life expectancy is an indicator for this measurement.

## Developments in Healthy Life Expectancy Research

Healthy life expectancy is an indicator that represents a composite of data on mortality and health status and is defined as the average number of years that a person can expect to live at a certain level of health.

The concept of healthy life expectancy was first proposed by Saunders in the United States in the 1960s and was first calculated by Sullivan in the 1970s. With further advancements in the methodology, it began to be utilized worldwide in the late 1980s. In 2000, the World Health Organization (WHO) calculated the healthy life expectancy of all its Member States. In the Health Japan 21 initiative of the Ministry of Health, Labor and Welfare (MHLW) of Japan, which was launched in 2000, the extension of healthy life expectancy was listed as one of the most urgent goals.

The concept of healthy life expectancy is further categorized into disability-free life expectancy (DFLE) and health-adjusted life expectancy (HALE). DFLE is calculated by taking the average number of years in which a person is expected to live without being in unhealthy status (e.g., limited activity level, poor subjective health status, need for long-term care, dementia, need for care in a long-term care facility), whereas HALE is calculated by assigning weights to various kinds and severity of unhealthy status for each individual ^(2)^.

The WHO uses HALE as an indicator of healthy life expectancy, whereas Japan and most Western countries use DFLE. In the Health Japan 21 initiative (second term) of the MHLW, healthy life expectancy is defined as the average number of years a person is expected to live without any limitations on his or her daily activities, in which the term “daily activities” represents a broad concept that encompasses activities of daily living (ADL), going out, working/doing housework/studying, and exercise and sports ^(2)^.

In 1993, wemeasured the healthy life expectancy (defined as the average life-years without requiring assistance for ADL) of residents in Sendai city and reported that, at age 65 years, healthy life expectancy was 14.7 years for men and 17.7 years for women ^(2)^. Because life expectancy at age 65 years was estimated to be 16.1 years for men and 20.4 years for women, the number of life-years to be spent with ADL disability was 1.4 years for men and 2.7 years for women. In short, women would live with ADL disability for twice as long as men, although women live longer than men ^(3)^.

We further compared these values with those reported for the elderly in the United States ^(4)^ (Table 1). Healthy life expectancy among the Japanese subjects was longer than those among the American elderly. The percentage of healthy life expectancy to total life expectancy (% Healthy Life) was comparable with those in the United States. Thus, we suggested that Japan was better in terms of functional status among the elderly, although there is some difficulty in comparing self-reported disabilities across cultures. Later, a report from the WHO in 2000 confirmed that the Japanese population has the longest healthy life expectancy in the world.

**Table 1.** Comparison of Total and Healthy Life Expectancies at Age 65 Years between Japan and the United States.

[Men]					
	Total	Healthy life expectancy	Life-years with ADL disability	% Healthy life
Sendai	16.1 yr	14.7 yr	1.4 yr	91.3%
East Boston	11.9 yr	10.6 yr	1.3 yr	89.1%
Iowa	15.5 yr	12.3 yr	3.0 yr	80.0%
New Haven	12.6 yr	10.4 yr	1.2 yr	82.5%
	
[Women]					
	Total	Healthy life expectancy	Life-years with ADL disability	% Healthy life
Sendai	20.4 yr	17.7 yr	2.7 yr	86.8%
East Boston	16.3 yr	14.4 yr	1.9 yr	88.3%
Iowa	20.5 yr	16.7 yr	3.8 yr	81.5%
New Haven	19.1 yr	15.8 yr	3.3 yr	82.7%

(Based on Tsuji I, Minami Y, Hisamichi S, et al. Active life expectancy among elderly Japanese. J Gerontol. 1995;50A:M173-6 ^(3)^ and Branch LG, Guralnik JM, Doley DJ, et al. Active life expectancy for 10,000 Caucasian men and women in three communities. J Gerontol. 1991;46:M145-50 ^(4)^.)

## Observational Studies on Factors Associated with Healthy Life Expectancy

To identify factors that affect healthy life expectancy, we performed cohort studies in the aged population of Miyagi Prefecture (Ohsaki city and Sendai city), which have been approved by the Ethics Committee of Tohoku University Graduate School of Medicine. We have found the following:

- Regarding blood pressure, we performed a cohort study to examine the association between blood pressure measured at home and mortality risk. In this study, we were the first in the world to propose the reference values of blood pressure measured at home as 137/84 mmHg, which represented the lowest risk of mortality ^(5)^.
- Regarding serum epidemiology, we demonstrated that individuals with higher serum equol and lower serum adiponectin levels were at a lower risk of mortality and less likely to develop ADL disability requiring certification of long-term care insurance ^(6)^.
- Regarding lifestyle habits, we demonstrated that individuals who drank green tea more frequently had a lower risk of mortality from cardiovascular disease and myocardial infarction ^(7)^ and that individuals who followed a Japanese dietary pattern more closely were less likely to develop ADL disability requiring certification of long-term care insurance, develop dementia, or die from cardiovascular disease ^(8)^. We also demonstrated that healthy life expectancy (defined as the average life-years without requiring certification of long-term care insurance) of individuals who met the three criteria for healthy lifestyle habits (nonsmoking, walking ≥ 30 min/day, and ≥ 270 g of fruit and vegetable intake/day) was 17.1 months longer than that of those who met only one or none of these criteria ^(9)^.
- Regarding psychological and social factors, we demonstrated that individuals who feel *ikigai* (a sense of life worth living) and actively participated in social activities were at a lower risk of mortality and less likely to develop ADL disability requiring certification of long-term care insurance ^(10)^.

## Intervention Studies on Extending Healthy Life Expectancy

We conducted intervention studies in the aged population, primarily in Sendai city, to examine the effectiveness of intervention measures for extending healthy life expectancy. These studies have been approved by the Ethics Committee of Tohoku University Graduate School of Medicine. We have demonstrated the following:

- For the first time in Japan, we conducted a randomized controlled trial to demonstrate the effectiveness of aerobic exercise training among the aged individuals ^(11)^.
- We performed a comprehensive geriatric assessment that aimed to help prevent residents aged ≥70 years from developing disability. In that study, we identified individuals who were at high risk of motor and cognitive function decline, depression, and poor oral hygiene, and examined the effectiveness of preventive interventions (the Tsurugaya project) ^(12)^. That study provided evidence that helped in the development of the long-term care prevention program implemented by the MHLW in 2004.
- We examined the outcomes of the long-term care prevention program in several municipalities of Miyagi Prefecture. This program included initiatives to improve nutritional intake and dental oral care. We compared the risk of developing ADL disability requiring certification of long-term care insurance between the program participants and the nonparticipants by propensity score matching and demonstrated that the program was effective in reducing the risk of developing ADL disability ^(13)^.

## Policy Proposals for Extending Healthy Life Expectancy

Based on the evidence generated from previous studies, I made policy recommendations aimed to extend healthy life expectancy. In particular, I was involved in the development of long-term care prevention measures and of a basic checklist (*kihon checklist* in Japanese) as the Chair of the Evaluation and Research Committee on Long-term Care Prevention Servicesof MHLW in 2004. As the Chair of the Planning Committee for the Next National Health Promotion of MHLW, I contributed to the development of the Health Japan 21 (second term) in 2011. In the book entitled “Achieving Healthy Longevity Society,” which was published in 2015 by Taishukan Publishing Co., Ltd., I proposed a comprehensive approach to extend healthy life expectancy ^(14)^.

In working with the government to develop the “Healthy Life Expectancy Extension Plan,” I provided supporting evidence for the healthy life expectancy targets for 2040 (extension of healthy life expectancy by at least 3 years relative to 2016 in both men and women and a healthy life expectancy of at least 75 years for both men and women) in the conference mentioned previously ^(2)^.

## Aiming for Compression of Morbidity

I first learned the concept of healthy life expectancy when I was studying in the United States in 1991. I was deeply moved after reading the manuscript entitled “Active Life Expectancy”^(15)^ written by Dr. Sidney Katz. Also, the manuscript entitled “Compression of Morbidity”^(16)^ written by Dr. James Fries determined my lifework to make compression of morbidity come true.

Compression of morbidity refers to the idea that we should focus on the duration to be spent in an unhealthy state, which is the difference between total and healthy life expectancies. With extension of life expectancy, it is important how healthy life expectancy extends. When Dr. Fries published the above paper, most researchers considered that people would live longer and sicker; thus, the duration to be spent in an unhealthy state would be increasing with extending total life expectancy. Dr. Fries proposed, however, that the duration to be spent in an unhealthy state can be compressed if healthy life expectancy extended more than total life expectancy did. If compression of morbidity comes true, it is expected that health status and quality of life at the individual level would improve, and the social security burden and economic productivity at the societal level would reduce. This is certainly an ideal scenario. As such, Dr. Fries suggested that compression of morbidity is the most critical goal to be targeted in medical practice today and would come true by delaying the onset of chronic illnesses through further health promotion and disease prevention efforts. When I read the paper by Dr. Fries, I decided that, as a public health researcher and practitioner, I should devote my life to achieving compression of morbidity.

Looking back, my research has always focused on compression of morbidity. To achieve this, I have collected various types of evidence through epidemiologic studies. In 2011, as the Chair of the Planning Committee for the Next National Health Promotion of MHLW, I proposed that the Health Japan 21 (second term) should aim to extend healthy life expectancy to exceed the number of years extended in the total life expectancy. This in fact is the realization of compression of morbidity.

**Figure 1.**
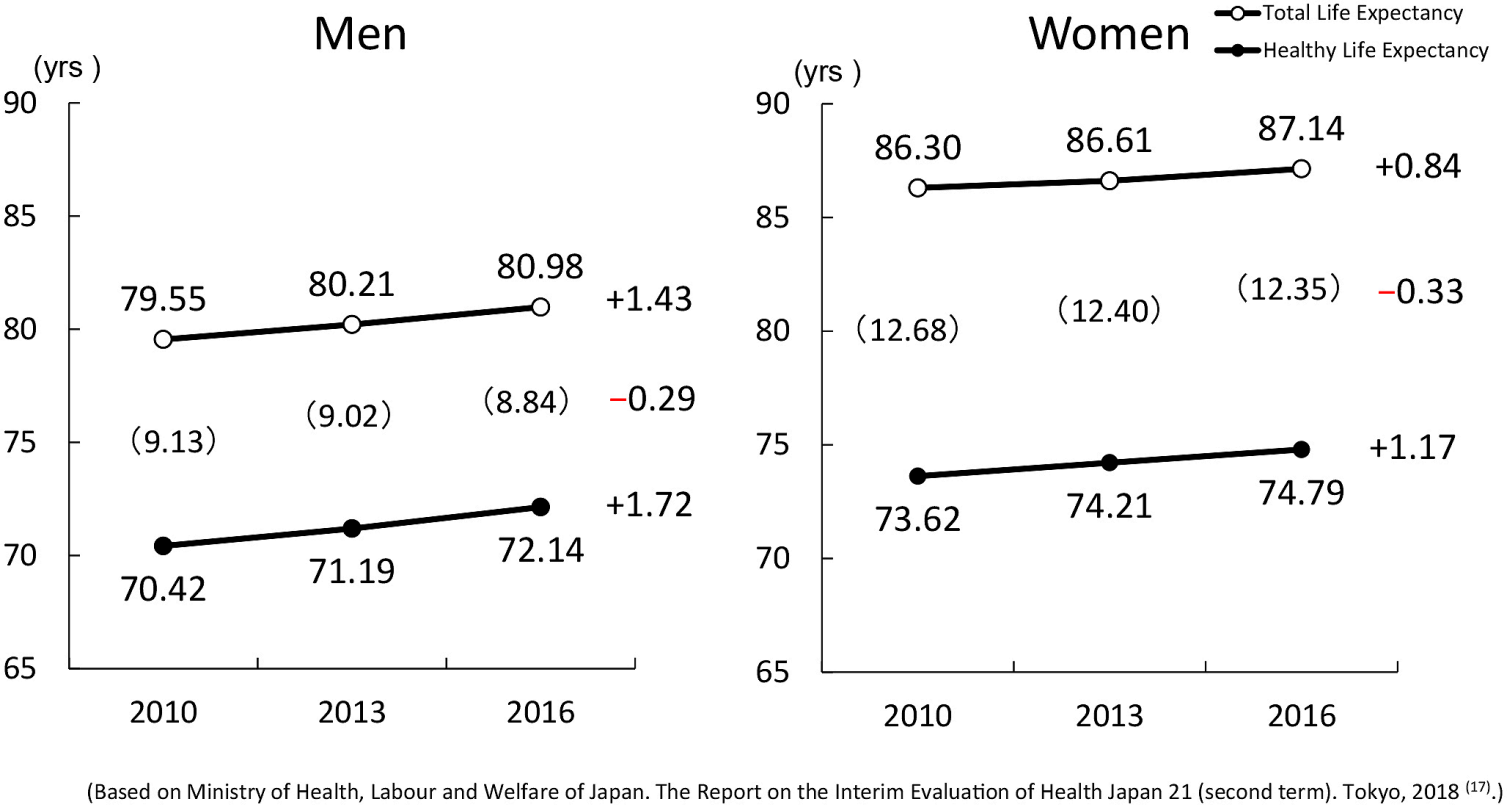
Trends of total life expectancy and healthy life expectancy in Japan.

In the interim evaluation of the Health Japan 21 (second term) in 2018, we were able to demonstrate that this goal is being achieved. Compared with 2010, in 2016, healthy life expectancy had been extended by 1.72 years in men and by 1.17 years in women. During the same time period, total life expectancy increased by 1.43 years in men and by 0.84 years in women. Therefore, the increase in healthy life expectancy was higher than that of total life expectancy. As a result, the duration to be spent in an unhealthy state was reduced by 0.29 years in men and by 0.33 years in women (Figure 1) ^(17)^.

The reduction by approximately 0.3 years within 6 years in the duration to be spent in an unhealthy state may be relatively small. However, it is important to note that Japan is the only country that has made progress in achieving compression of morbidity at the national level. Therefore, even a 0.3-year reduction is significant and should not be underestimated. This is a good starting point to continue the efforts to compress the duration to be spent in an unhealthy state.

I am blessed to have been able to pursue the research theme that really touched me as I was starting out as a researcher and to have been involved in the process of evidence generation and policy development to see the implementation of the end product. I am grateful to every individual who helped me achieve our goals along the way.

However, compression of morbidity has just started, and we need to maintain this momentum. I sincerely appreciate the guidance of and support from the members of the Japan Medical Association.

## Article Information

### 

This article is based on the study, which received the Medical Award of The Japan Medical Association in 2019.

This is a revised English version of the article originally published in Japanese in the Journal of the Japan Medical Association 2019;148(9):1781-4 ^(18)^. The original version is available at https://www.med.or.jp/cme/jjma/newmag/14809/14809.html.

The Editors-in-Chief of the Journal of the Japan Medical Association and JMA Journal have permitted the publication of this manuscript.

### Conflicts of Interest

None
